# Sonographic guided hydrostatic saline enema reduction of childhood intussusception: a prospective study

**DOI:** 10.1186/s12873-018-0196-z

**Published:** 2018-11-21

**Authors:** Ademola Olusegun Talabi, Olusola Comfort Famurewa, Kayode Taiwo Bamigbola, Oludayo Adedapo Sowande, Babalola Ishmael Afolabi, Olusanya Adejuyigbe

**Affiliations:** 1 Department of Surgery, Obafemi Awolowo University, P. O. BOX 5538, Ile-Ife, Osun State Nigeria; 2 Department of Radiology, Obafemi Awolowo University, Ile-Ife, Osun State Nigeria; 3 Department of Surgery, Federal Medical Centre, Owo, Ondo State Nigeria

**Keywords:** Childhood intussusception, Hydrostatic reduction, Saline, Ultrasound-guided

## Abstract

**Background:**

The management of childhood intussusception in our sub-region is still via surgical intervention. Currently, the gold standard of treatment is non-operative reduction. We sought to assess the suitability of hydrostatic (saline) reduction of intussusception in children in our institution.

**Materials and methods:**

A prospective study was conducted between January 2016 and June 2017 in all children with ultrasound confirmed intussusception at a tertiary teaching hospital in Nigeria. All children excluding those with signs of peritonitis, bowel gangrene and intestinal prolapse were selected for ultrasound-guided hydrostatic reduction (USGHR). We allowed a maximum of three attempts at reduction.

**Results:**

The age range was 3 months to 48 months with a mean of 10.8 ± 9.1 months. Forty percent (*N* = 18) presented after 24 h of onset of symptoms. The success rate of hydrostatic reduction with saline enema was 84.4% (*N* = 38). Two (4.4%) perforations occurred during the procedure. Three (7.5%) patients had recurrent intussusception within six months. The duration of symptoms greater than 24 h, age and sex of patients did not influence successful reduction *p* > 0.05. The duration of admission between those who had successful non-operative reduction and those who subsequently had operative reduction and or resection attained statistical significant difference, *p* = 0.001. There was no mortality. We achieved a 68% decrease in the operative reduction of intussusception using USGHR as the primary modality of treatment.

**Conclusion:**

Our study found out that USGHR is a suitable alternative for the treatment of childhood intussusception.

## Background

Intussusception is a common surgical emergency in infants and toddlers. It is also seen in older children and in adults occasionally. The incidence of intussusception is approximately one to four per 2000 infants and children [1, 2]. Most (90%) of the intussusception are ileocolic, while the remaining 10% are of the ileoileal or colocolic type [3].

The treatment modality in our environment is still open surgery due to late presentation of patients, misdiagnosis from peripheral health centres, dearth of modern imaging equipment such as fluoroscopic machines, and in some cases lack of expertise to undertake non-operative reduction of intussusception [1, 4].

Hydrostatic reduction under ultrasound guidance is a well-recognized alternative method for reduction of childhood intussusception [5]. Kim et al. [6] described the first successful sonographic guided hydrostatic reduction of intussusception in 1982. Since then, there has been widespread use of this technique due to less morbidity and mortality compared with surgical form of treatment [7]. The other non-surgical methods are reduction with barium or air under fluoroscopic guidance [5, 8]. These other non-surgical methods of reduction under fluoroscopy are either non-existent or dysfunctional in most centres in sub Saharan Africa including our hospital [1, 9], thereby making ultrasound guided reduction of intussusception a better modality of treatment in resource poor environment. Ultrasound scanners are relatively cheap and readily available in most hospitals thereby rendering ultrasound guided hydrostatic reduction (USGHR) cost effective for patients. The main advantage of ultrasound guided hydrostatic reduction over reduction under fluoroscopic guidance is the avoidance of exposing young children to ionizing radiation. As ultrasound is often the first – line-imaging modality for the diagnosis of intussusception, the procedure can be performed within the ultrasound room immediately after the diagnosis is made [5, 8]. The other benefits of ultrasound guided hydrostatic reduction include less patient discomfort, shorter hospital stay, and less morbidity and mortality compared to surgical modality of treatment.

The various forms of enema in use for ultrasound guided liquid enema in use include portable tap water, normal saline or Ringers lactate solution [8, 10, 11]. In a review by Bekdash et al. [12], the overall success rate of non-operative reduction of intussusception ranged from 46 to 94%, while recent studies reported that the success rate for hydrostatic reduction with saline ranges from 55.6 to 90% [1, 13, 14]. A much more recent study from Ethiopia found a successful reduction rate of 87.2% [9].

Prior to this time, laparotomy was the only treatment option available for all cases of intussusception in our hospital with unacceptably high mortality rate of 12 to 15.4% [4, 15]. Subsequently, we conducted a pilot study of five consecutive children with intussusception who presented with no signs of peritonitis and other features that could preclude non-operative reduction with successful non operative reduction in all by using normal saline under ultrasound guidance.

Following our anecdotal success, we decided to conduct this study to assess the suitability of normal saline hydrostatic reduction of intussusception among children with intussusception irrespective of the age and duration of symptoms provided they meet the inclusion criteria set for non-operative reduction of intussusception in our hospital.

## Methods

### Design

This was a prospective cross sectional study of all children treated between January 2016 and June 2017 at a tertiary hospital in Nigeria.

### Setting

Our institution is situated in south west, Nigeria where it provides primary and secondary health care services in addition to its main tertiary care to people who are mainly Yorubas. Majority of our population are farmers, artisans and civil servants. The paediatric surgical unit of our hospital was established in 1980 and has recently been upgraded to a 60-bedded facility to cater for children less than 16 years.

### Subjects and inclusion/exclusion criteria

The ethics and research committee of our hospital approved the study. Patients who presented to the children emergency unit of our hospital were recruited into the study. All parents or guidance of the patients with suspected intussusception were informed about the procedure and they signed written informed consent to participate in the study in their local language or in English. All consecutive patients with suspected intussusception admitted via children emergency of our hospital were enrolled into the study. The inclusion criteria for the population of patients elected to undergo USGHR are all children with ultrasound-diagnosed intussusception. Exclusion criteria included the following: (1) clinical features of perforation and peritonitis. (2) Prolapsed intussusception.

Data retrieved from the case notes included age, sex, duration of symptoms, clinical features, results of treatment, surgical and pathological findings if the patients underwent subsequent laparotomy, complications, and follow-up after discharge. In addition, we collected samples for packed cell volume, electrolytes and cross matching of blood. Other variables collected included the time required to perform the USGHR, number of times procedure was carried out, the volume of fluid used to achieve reduction as well as duration of admission.

### Outcome measures and measurement

The outcome measures were successful reduction, failed reduction with subsequent surgical intervention, perforation during reduction and recurrence of intussusception after treatment.

We defined successful reduction as one in which there was complete disappearance of the intussusceptum with reflux of saline into the ileum.

Failed reduction was defined as one in which the intussusceptum could not be reduced completely or there was perforation of the gut.

Late presentations were those presenting after 24 h of abdominal pain.

### Procedure

All patients were placed on intravenous infusion of 4.3Dextrose in 1/5 normal saline for hydration, nil per os, intravenous cefuroxime and metronidazole and urinary catheter to prepare the patient for laparotomy in case reduction failed.

Ultrasound scan of the abdomen was performed in the radiology ultrasound suite by the radiology senior registrar with Mindray DC - 7 ultrasound machines with 10 - 12 MHz high frequency linear probe to confirm the diagnosis of intussusception. After confirmation, an attending radiologist and senior registrar in paediatric surgery performed USGHR. An on-call paediatric surgeon, anaesthetist and peri-operative nurses were informed prior to the procedure in case of complication such as perforation and failed reduction that require surgery.

The USGHR was performed in the following manner with the patient lying supine, an appropriate Foley catheter (10F – 18F) was inserted into the rectum and balloon inflated. No sedation was t administered as the patient was held in position by radiology staff. Normal saline, pre-warmed to body temperature was suspended 120 cm: above the table level (within the range as described by He et al. [16] and allowed to flow into the colon under gravity. During reduction, the retrograde flow of saline and the regress of intussusceptum were monitored under ultrasound visualization. In addition, the peritoneal cavity was scanned intermittently for the presence of sudden increase in fluid and simultaneous loss of fluid from the colon, indicating bowel perforation. A maximum of 3 attempts were allowed, with each reduction lasting 3 to 5 min with an interval of less than 3 min. If patients were still not reduced after the third attempt, the procedures were stopped immediately and they were taken to theatre immediately for surgical reduction or resection with bowel anastomosis. All patients who had undergone successful USGHR were kept in the ward for observation for at least 24 h to evaluate for complications and recurrence.

### Data analysis

Data collected were analyzed using Microsoft excel and Statistical Package for Social Scientists version 17 for windows (SPSS Inc. Illinois, Chicago, USA) The data were summarized using means and standard deviation (SD) for continuous variables and frequencies for categorical variables. Inferential statistics with Chi Square test was used to establish association with *p* value less than 0.05 considered as statistically significant.

## Results

During the study period, we managed 51 children with 56 intussusceptions. Eleven of the patients with eleven intussusceptions were excluded either due to features of peritonitis or intestinal prolapse and were taken to theatre for primary surgery. Only 40 patients with 45 intussusceptions who met the inclusion criteria were analysed. They comprised of 22 males and 18 females giving a male to female ratio of 1.2 to 1.

**Fig. 1:**
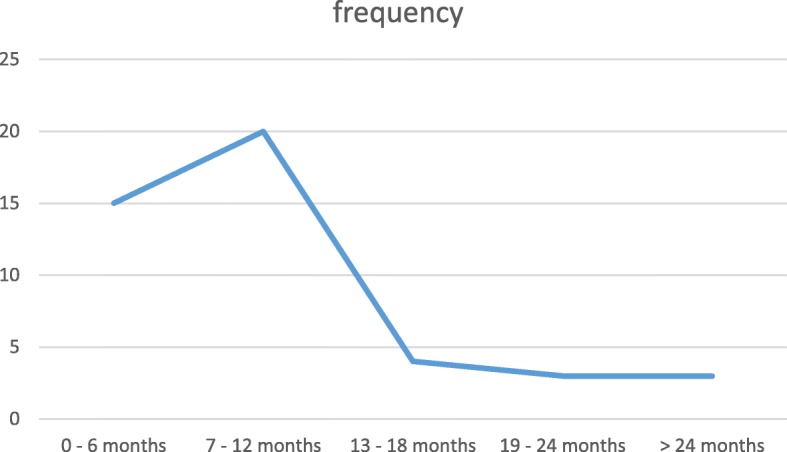
xxxxx

Their age ranged from 3 months to 48 months with a mean age of 10.8 ± 9.1 and a median of 7 months. Figure 1 shows the distribution of the age of occurrence of intussusception among patients. The peak age incidence was between 7 to 12 months. Most 60% (*N* = 27) patients presented within 24 h of onset of abdominal pain while 40% (*N* = 18) presented after 24 h.

The duration of symptoms ranged from 3 h to 144 h with a mean of 40.6 ± 36.1 h and a median of 24 h. The clinical features are as shown in Table 1, with the most common symptoms being colicky abdominal pain (100.0%), vomiting (100.0%), and palpable abdominal mass (95.6%).

**Table 1:** Clinical features of patients

Clinical Features	Frequency (%)
Colicky abdominal pain	45 (100%)
Vomiting	45 (100%)
Red currant stool	36 (80.0%)
Abdominal distention	6 (13.3%)
Palpable abdominal mass	43 (95.6%)
Dehydration	18 (40.0%)
Fever	14 (31.1%)

Thirty-eight of the intussusception (84.4%) were ileocolic,4 (8.9%) were colocolic while 3 (6.7%) were ileoileal intussusception. Table 2 shows the outcome of various types of intussusception. We found no pathologic lead point in our series.

**Table 2:** Outcome of various types of intussusceptions

Types of intussusception	successful	Non-successful
Ileoileal	2	1
Ileocolic	33	5
Colocolic	3	1

Thirty-eight (84.4%) cases were successfully reduced under ultrasound guidance while 7 (15.6%) cases had partial reduction or failed reduction of intussusceptum necessitating open surgery. Of the failed cases, 2 had bowel perforation during the procedure which was confirmed at surgery to be due to gangrenous bowel, 2 had gangrene of the bowel without perforation. The remaining three cases had hyperplasic Peyers patches with gross edema of the apex of the intussusceptum. Six patients had bowel resection while one patient had manual reduction. Hydrostatic reduction appears to be more successful in patients with shorter duration of symptoms but this was not statistically significant, *p* = 0.098, (Table 3).

**Table 3:** Factors affecting non-operative reduction of intussusception

Variables	Outcome of non-operative reduction		*P* value
	Non successful	Successful	
Duration of abdominal pain in hours			0.098
< 24	2 (7.4%)	25 (92.6%)	
> 24	5 (27.8%)	13 (72.2%)	
Age (months)			0.182
0–12	7 (20.0%)	28 (80.0%)	
13–48	0 (0.0%)	10 (100.0%)	
Gender			1.000
Male	4 (16%)	21 (84%)	
Female	3 (15%)	17 (85%)	

In addition, the age and gender did not influence successful reduction of intussusception, *p* > 0.05 (Table 3).

Three patients (7.5%) had recurrent intussusception during follow up. Two patients had 2 episodes of recurrence after the first intussusception at an interval of 3 to 6 months apart while the third patient had a single episode of recurrence within 24 h after initial reduction. These patients had successful reduction non-operatively. Oral contrast enhanced computerized tomographic scan of the abdomen done for the 2 patients with 2 recurrences revealed no pathology either on the wall or lumen of the intestine. Most (20/38) of the reduction was achieved during the first attempt of the procedure The duration of the procedure ranged between 3 min to 25 min, with a mean of 8.0 ± 5.5 min. The mean duration of admission between those who had successful reduction was 2.5 ± 0.6 days and those with failed reduction that subsequently had surgery was 9.1 ± 1.5 days. The difference attained statistical significance, *p* = 0.001. There was no mortality in our series.

There was a 68% (38/56) decrease in the operative reduction of intussusception following saline hydrostatic reduction under ultrasound guidance.

## Discussion

Non-operative reduction has been the gold standard of treatment of intussusception in developed countries. Non-operative treatment includes reduction with barium, air or saline enema under fluoroscopic or ultrasound guidance. Since the widespread acceptance of saline reduction under ultrasound guidance, published data indicated high success rates comparable to, or better than fluoroscopic barium or air reduction. The use of USGHR as mainstay of treatment has been slow to take place in many developing nations including Nigeria due to late presentation, misdiagnosis, either lack of or dysfunctional fluoroscopic units and lack of expertise to undertake the procedure in many hospitals [1, 4, 9]. The high mortality rate associated with surgical treatment in developing countries over its developed counterpart has underscored the need for more countries especially those in sub-Saharan Africa to embrace USGHR [4, 15]. There is need for many health professionals to have high index of suspicion to prevent misdiagnosis and public enlightenment on the part of the populace to prevent late presentation so that many patients can benefit from non-operative reduction. In this series, the rate of operative reduction of intussusception decreased by 68% which was comparable to what was obtained by Wakjira et al. in Ethiopia a developing nation like ours. This implies that health facilities in low resource nations should embrace this non-operative method of managing childhood intussusception.

In the current study, 84.4% of the reduction was successful. This was similar to the findings of other workers [3, 14, 17–19] where the success rate of ultrasound guide hydrostatic reduction was more than 82%, which was somewhat more than the success rate of 75% recorded by Mensah et al. in Ghana [8]. Ogundoyin et al. [1] in Nigeria found a lower successful reduction rate of 55.6%. Wakjira et al. [9] in a recent study were able to achieve 87.2% reduction rate. However, Sanchez et al. [20] in a subset of 14 children that underwent hydrostatic reduction with saline recorded 100% success rate.

Recurrence rate after non-operative reduction intussusception ranges from 5 to 20% with a mean of 10% [21]. Recurrent intussusception in which there is a pathologic lead point even has a higher incidence of recurrence in about 8 to 9% of cases. Half of recurrent intussusception usually occurs within 48 h but recurrences up to 1.5 years later have been documented [22–24]. In the present study, the recurrence rate was 7.5%, which was consistent with other literature reports [1, 8, 18, 25]. Gray et al. [26] in a meta-analysis of recurrence rate of non-operative reduction of intussusception found a recurrence rate of 7.5% with saline reduction of intussusception. Recurrent intussusception is amenable to treatment via USGHR, even if it occurs several times [21]. It is worth noting in this series that two children had two late recurrences each at an interval of 3 to 6 months apart with the last episodes being before the age of 18 months. Oral contrast enhanced computed tomography scan of the abdomen confirmed no pathology within or outside the intestine in these children. Non-operative reduction with saline under ultrasound guidance was successful in all recurrent cases in our series. This significant finding stresses the fact that most intussusception are idiopathic and that non operative reduction should be entertained in patients with several late recurrences provide they meet the inclusion criteria for this procedure.

This study finding has shown that age and sex of patients has no role to play in the success of hydrostatic reduction. Our study finding is in agreement with most reports [1, 16, 18]. However, Nayak et al. [17] observed a lower successful reduction in young infants. In the same vein, Eklof et al. [27] in a series of 658 radiologically diagnosed childhood intussusception reported a markedly reduced rate of successful reduction in infants compared with older children. They concluded that the ileocaecal valve for reasons unknown may be more competent in the very young, and makes it practically difficult to allow the flow of contrast into the terminal ileum infants.

The duration of symptoms is an important predictor of outcome of non-operative reduction of intussusception in children Wong et al. [28] found that a mean duration of symptoms of 2.3 days did not affect the success rate of reduction. In contrast, Chung et al. [29] studied the risk factors leading to surgical reduction and found that long-standing duration of symptoms (> 24 h) was a risk factor for failed reduction. Khorana et al. [25] concluded that the presence of intestinal viability rather the long duration of symptoms is an important risk factor for failed reduction. In our series as in some reports [17, 18, 25, 30] the duration of symptoms did not influence successful reduction of intussusception.

The incidence of intestinal perforation during USGHR appears to be low ranging from 0 to 10% in some series [1, 3, 4, 19]. Bowel perforation due to over insufflation with fluid is a risk but most cases of perforation with reduction are said to have occurred before the procedure and as such, these are ‘unavoidable’ [17] Most of the perforation occurring during the procedure are due to intestinal gangrene rather than high intraluminal pressure from saline. In our present study as experienced by some researchers [8, 9] two patients had intestinal perforations during the procedure. These two cases at laparotomy had bowel gangrene, which was ‘missed’ during clinical evaluation of the patients. This important finding has underlined the need to careful selection of patients clinically combined with the use of color Doppler ultrasound to assess the vasculature of the bowel prior to reduction. Nevertheless, intestinal perforation due to over inflation or ‘missed’ bowel gangrene should not discourage the use of sonographic guided hydrostatic reduction of intussusception in resource constraint hospitals where there are no facilities for hydrostatic pressure control.

Some studies [6, 31] recorded higher success rate when children were premedicated with chlorpromazine prior to hydrostatic reduction. Flaum et al. [18] found a positive correlation between the use of sedatives and high rate of successful reduction. Bia et al. [32] premedicated all children in their series with wintermin (1 mg/kg) and recorded up to 96% success rate. Mensah et al. [8] in Ghana found a success rate of 75% despite the use of 1 – 2 mg/kg of ketamine hydrochloride. We did not give sedatives to the patients in our series. The success rate could not have been better in our study if sedatives were given because majority of those with failed reduction had gangrenous bowel before presentation at our hospital.

## Conclusions

Hydrostatic normal saline enema reduction of intussusception under real time ultrasound is a suitable non-operative technique of managing childhood intussusception with a success rate of 84, 4% in our study. The approach is simple safe and cost effective in a resource constraint environment. We recommend its adoption as the standard technique for managing childhood intussusception in health care centres where facilities and expertise are available.

### Limitation

Our study has some limitations. Most notably was our small size of 51 children with 56 intussusceptions. We had relatively little experience in performing USGHR in our centre.

## Abbreviations

cm: Centimeter
DC-7: Colour Doppler 7
F: French gauge
Mg/kg: Milligram per kilogram body weight
MHz: Mega Hertz
N: Frequency
SD: Standard deviation
SPSS: Statistical package for social sciences
USGHR: Ultrasound guided hydrostatic reduction
